# Supplementary material for: How transfer flights shape the structure of the airline network
Source: Sci Rep. 2017 Jul 17;7:5630. doi: 10.1038/s41598-017-06108-z (PMC5514031; doi:10.1038/s41598-017-06108-z)
Supplement: Supplementary file 1 — Supplementary Material [file 41598_2017_6108_MOESM1_ESM.pdf]

How transfer flights shape the structure of the airline network

Tomasz Ryczkowski, Agata Fronczak, Piotr Fronczak

Supplementary material

GDP vs population size in the gravity equation

In the paper, we have assumed that the flow between the two countries given by the gravity equation, Eq. (1), is directly proportional to their GDPs. One may equally think of using the population size as the mass term instead, for example. In Fig. S1 we show that there is a linear correlation between these two quantities. Thus, one may expect that replacing GDP by population size will not change the observed discrepancies between the real flow and the one modeled by the gravity equation. Fig. S2 confirms that expectation.

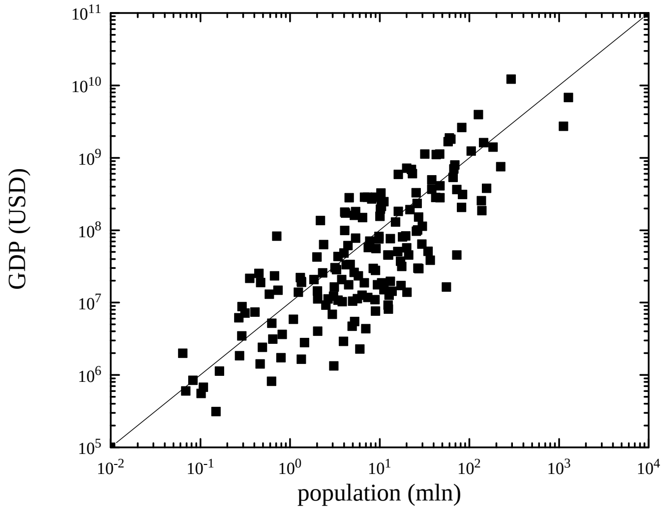

Figure S1. Linear depencence of GDP on population for all countries in year 2004.

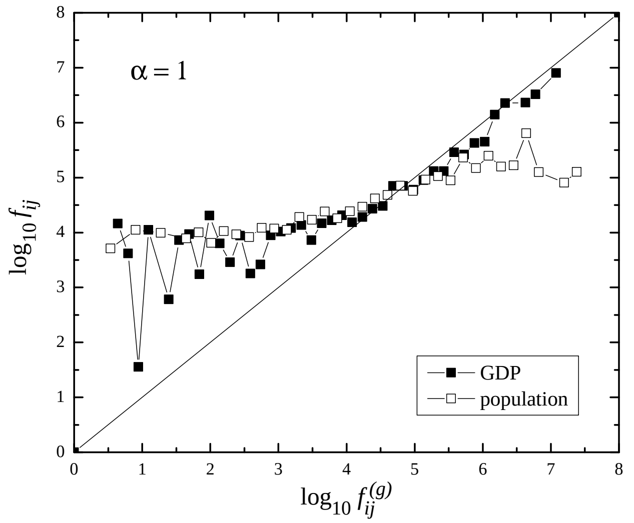

Figure S2. The observed weights of connections in the airline network, f_{ij}, vs. their expected values, $f_{ij}^{(g)}$ in year 2004. All data are logarithmically binned. Black squares correspond to those presented in Fig.1e and have been calculated using GDP as a mass term in Eq. (1). Open squares are based on Eq. (1) with the population size as a mass term.

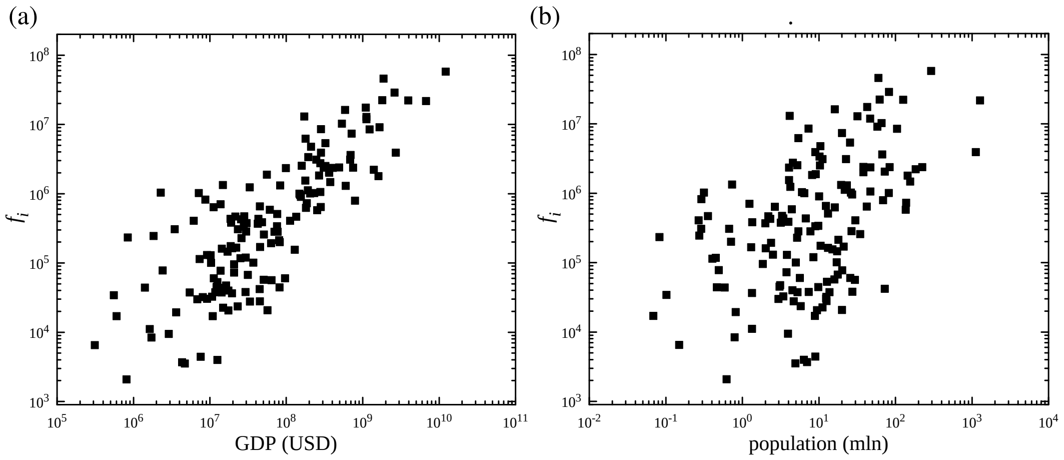

Figure S3. Total number of passenger flying from a given country vs its (a) GDP (b) population size, in year 2004.

The reason why we have decided to relay on GDP instead of population size is presented in Fig. S3. We show here the dependence of passenger volume on GDP (Fig. S3a) and population size (Fig. S3b) respectively. By the volume we understand the total number of passengers flying from a given country in year 2004. As one can see, the population-based dependence is much more diffused. Thus, one can expect that the modeling from such noisy data will give less convincing results.

Discussssion of the function $f(r_{ij}, r_{jk})$ in Eq. (11)

Below we discuss the impact of the choice of the function $f(r_{ij}, r_{jk})$ in Eq. (11) on the obtained results. Let us imagine the two countries i and k (the origin and the destination of a travel, respectively) that lie on the two dimensional plane at points $(x,y)_i = (0,0)$ and $(x,y)_k = (1,0)$ with no direct connection between them. Let us consider the two unnormalized probabilities of the choice of the intermediate stop j that have been mentioned in the main text, namely

$$f_1(r_{ik}, r_{jk}) = \frac{1}{r_{ij} r_{jk}}, \tag{1}$$

and

$$f_2(r_{ik}, r_{jk}) = \frac{1}{r_{ij}} + \frac{1}{r_{jk}}. \tag{2}$$

The both probabilities are presented in Fig. S4a and S4b. As one can see, they reflect the tendency of the passengers to choose

- the shortest paths $i \to j \to k$
- and the transfer airport j near to the origin or the destination country (i.e. the tendency to fly from the starting airport i to a nearby hub j that has many long distant connections or to fly from the starting airport i to a hub close to the destination airport k.).

The both probabilities are very similar, what is confirmed in Fig. S4c and S4d, that present the cross sections of the two-dimensional functions $f(x,y)$ through the axis $y = 0$ (Fig. S4c) and $x = 0$ (Fig. S4d). Such small differences between the both functions cannot lead to different final results. It is indeed confirmed is Fig. S5, which presents the comparison of the performance of the model of connected flights with the two discussed choices of probabilities $f(r_{ij}, r_{jk})$.

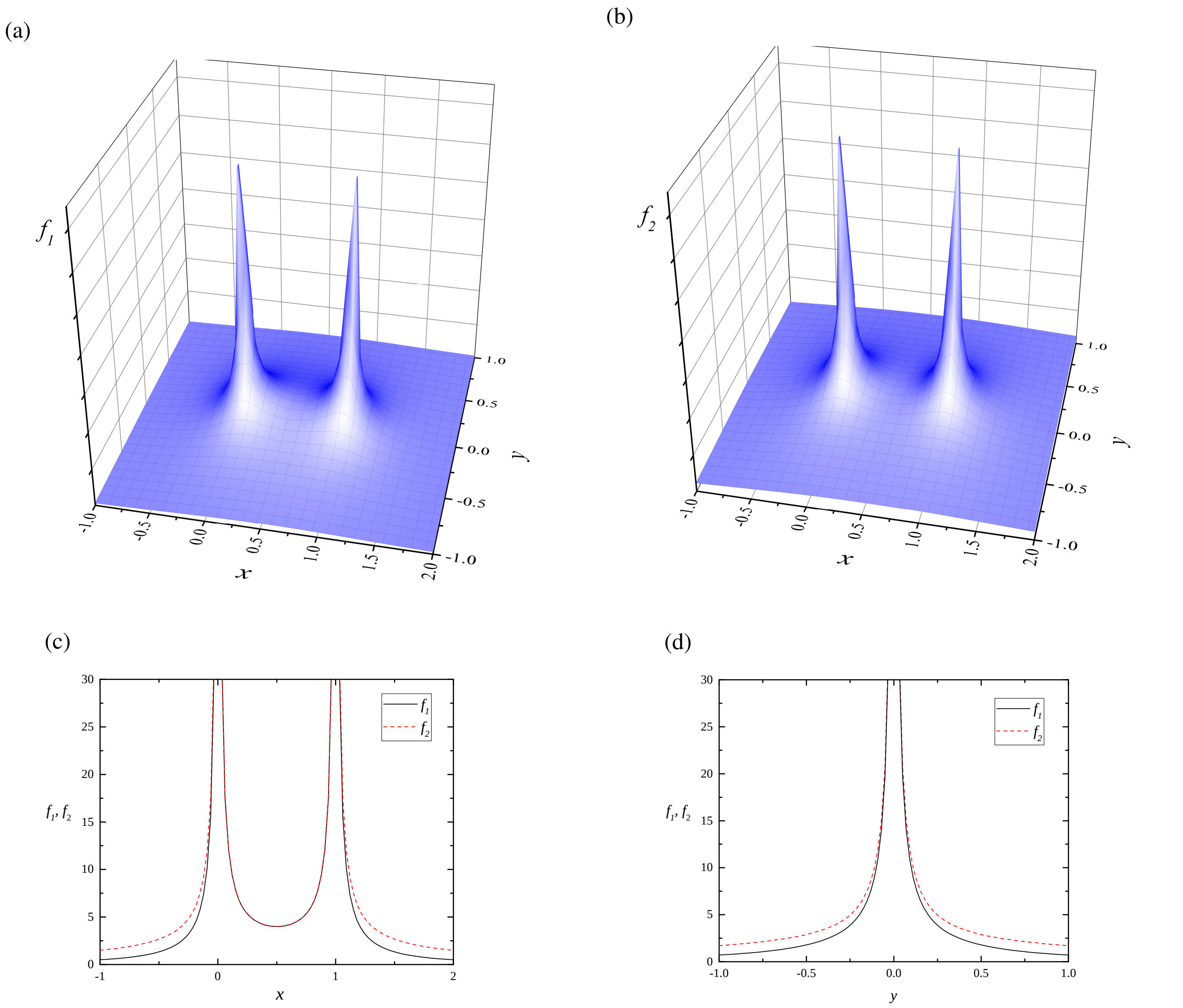

Figure S4. Unnormalized probabilities $f(r_{ij}, r_{jk})$ given by Eq. (S1) - (a) and (S2) - (b) and their two cross sections through the axis $y = 0$ (c) and $x = 0$ (d).

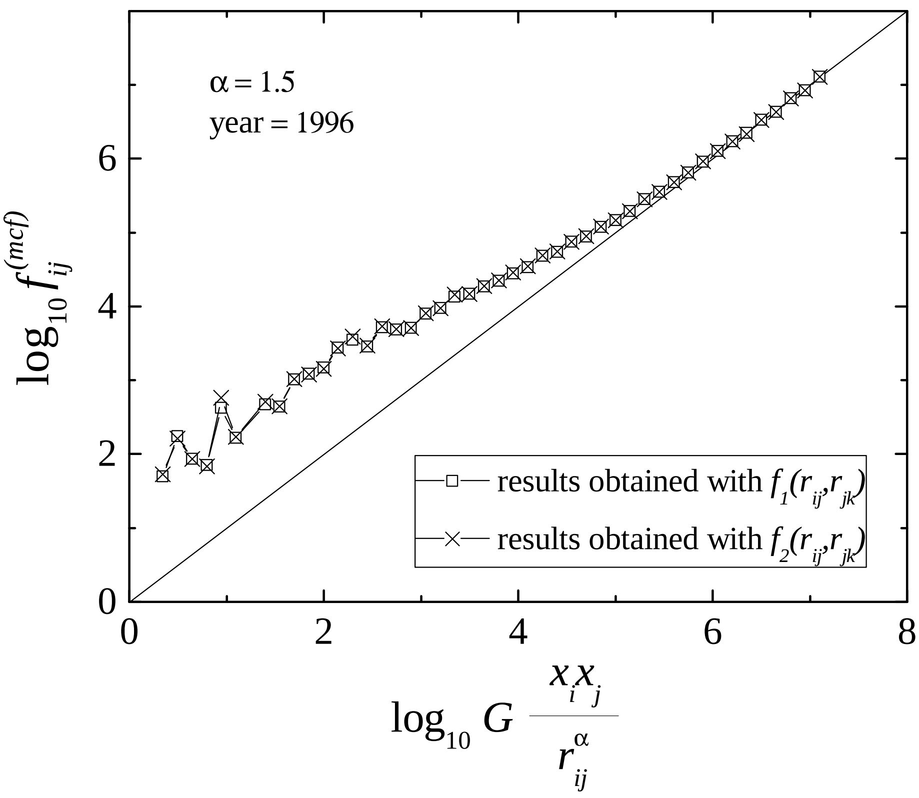

Figure S5. Comparison of the performance of the model of connected flights for year 1996 with the two discussed choices of probabilities $f(r_{ij}, r_{jk})$. Straight line corresponds to the standard gravity model.
